# Integrated DEA Models and Grey System Theory to Evaluate Past-to-Future Performance: A Case of Indian Electricity Industry

**DOI:** 10.1155/2015/638710

**Published:** 2015-03-04

**Authors:** Chia-Nan Wang, Nhu-Ty Nguyen, Thanh-Tuyen Tran

**Affiliations:** ^1^Department of Industrial Engineering and Management, National Kaohsiung University of Applied Sciences, No. 415 JianGong Road, Sanmin District, Kaohsiung City 807, Taiwan; ^2^International Relations Office, Lac Hong University, No. 10 Huynh Van Nghe, Bien Hoa, Dong Nai, Vietnam

## Abstract

The growth of economy and population together with the higher demand in energy has created many concerns for the Indian electricity industry whose capacity is at 211 gigawatts mostly in coal-fired plants. Due to insufficient fuel supply, India suffers from a shortage of electricity generation, leading to rolling blackouts; thus, performance evaluation and ranking the industry turn into significant issues. By this study, we expect to evaluate the rankings of these companies under control of the Ministry of Power. Also, this research would like to test if there are any significant differences between the two DEA models: Malmquist nonradial and Malmquist radial. Then, one advance model of MPI would be chosen to see these companies' performance in recent years and next few years by using forecasting results of Grey system theory. Totally, the realistic data 14 are considered to be in this evaluation after the strict selection from the whole industry. The results found that all companies have not shown many abrupt changes on their scores, and it is always not consistently good or consistently standing out, which demonstrated the high applicable usability of the integrated methods. This integrated numerical research gives a better “past-present-future” insights into performance evaluation in Indian electricity industry.

## 1. Introduction

According to U.S. Energy Information Administration (EIA), International Energy Statistics, and Oxford Economics, India was only trailing the United States, China, and Russia in energy consuming in the world. India used to be one of the ten largest economies in the world in 2012 and the third in gross domestic product (GDP) adjusted for inflation and purchasing power. This inflation-adjusted GDP has grown at over 7% per year since 2000, although it slowed to just over 5% in 2012 according to the Indian Central Statistical Organization. As a result, the growth rate for total energy consumption likely fell from prior-year levels. GDP, however, is expected to grow more and more in 2013 and 2014, and then EIA forecasts the greater energy consumption together with this growth of society [1].

Grover and Chandra [2] stated that India's energy policy is focused on securing adequate energy resources to meet the growing demands of its economy. The consumption of energy in India over doubled in the years from 1990 to 2011, especially primary energy. However, the sources of energy, which are imported, and its inconsistent energy sector reform, make it difficult to satisfy rising demand. Despite its growing energy use, India's per capita energy consumption remains much lower than that of developed countries, such as the United States. Other aspects of the Indian energy industry include the following.

India has 20 operational nuclear reactors, with seven more under construction [3]; as electricity demand continues to grow, India plans to increase its nuclear share of generation to 25%, up from 4% in 2011 [4].

In addition, the roles of the companies in the industry of electricity are generating, transmission, distributing, and/or any other activities that make people satisfied with the electricity quality and their demands in consumption. This entry consists of total electricity generated annually plus imports and minus exports, expressed in kilowatt-hours [5]. The discrepancy between the amount of electricity generated and/or imported and the amount consumed and/or exported is accounted for as loss in transmission and distribution [6].

Therefore, we need more new findings, efficiency, and effectiveness in the field of energy consuming or electricity in India. Moreover, performance evaluation is the important approach for enterprises to give incentive and restraint to their operators and it is also an important channel for enterprise stakeholders to get the performance information [7].

In this research, we will provide some insights after getting combined results of Data Envelopment Analysis (DEA) and Grey systems theory. Grey systems theory, which was first introduced by Deng [8], is a useful tool for accurate forecasting. Using DEA methodologies, we input some performance attributes and classify them as inputs and outputs and then use them for DEA researches. For ranking industries, we have used super-SBM method and followed it by Malmquist nonradial and radial for measuring the efficiency change, technical change, and productivity indices over the 5-year time frame from March 2010 to March 2014 (latest year) and next 4 years (2015–2018) of forecasting.

By this study, we expect to evaluate the rankings of the current Indian electricity companies under control of the Ministry of Power. In the meantime, this research would like to test whether any significant differences exist between two MPI models: Malmquist nonradial and radial. Then, we would choose one advance model of MPI to see these companies' performance in recent years and some next few years.

## 2. Literature Review

Filippini and Pachauri [9] stated that in order to understand the extent to which factors like income, prices, household size, and other household specific characteristics influence variations observed in individual households' electricity demand the results show that electricity demand is income and price inelastic in all three seasons and that household, demographic, and geographical variables are significant in determining electricity demand.

The power sector restructuring process initiated during 1991 has not succeeded in improving technical efficiency or in improving financial position of the power sector. Also, it could not reduce the losses or improve customer satisfaction. The social objectives of the power sector also could not be fulfilled effectively in the reform process. It is appropriate that serious review be made on the past performance of the power sector and effective steps taken [10].

Performance of 26 utilities was evaluated using the nonparametric technique of Data Envelopment Analysis (DEA), and the impact of scale on the efficiency scores was also evaluated. The results indicate that the performance of several SOEUs is suboptimal, suggesting the potential for significant cost reductions. Separate benchmarks were derived for possible reductions in employees' number, and the results indicate that several utilities deploy a much larger number of employees than that required by a best practice utility, and significant savings are possible on this account. Thakur et al. [11] argued that it was also found that the bigger utilities display greater inefficiencies and have distinct scale inefficiencies.

Zhou et al. [12] found that benchmarking of electricity utilities accounts for the largest number of studies, which is followed by the areas of modeling environmental performance and energy efficiency study. In the methodological aspect, they also found that the CRS reference technology and the radial efficiency measures are still the most widely used specifications. When both desirable outputs and undesirable outputs are considered simultaneously, it was found that the incorporation of environmental DEA technology with DDF efficiency measure could be a good choice. In addition, there has been a growing interest on the use of nonparametric MPI in E&E studies in recent years.

The super-SBM and Malmquist models were adopted by Lo and Lu [13], and they have argued the reasons for using DEA models. According to them, the SBM deals directly with input excesses and output shortfalls (slacks). The SBM reports an efficiency measure between 0 and 1 and gives an efficiency score of one if and only if the DMU concerned is on the frontier of the production possibility set with no input/output slacks. Intertemporal efficiency change, which is decomposed into “catch-up” and “frontier-shift” effects, is analyzed by means of the SBM-based Malmquist index. On the other hand, the super-efficiency-SBM is particularly useful in distinguishing efficient DMUs when the number of DMUs is small compared with the number of evaluation criteria [14].

Grey system theory was presented in 1982 by Julong Deng, Professor of Huazhong University of Science and Technology in Mainland China [15]. About the related researches, Huang and Yu [16] proposed several Grey-based models to forecast the monthly temperatures for two different cities. An et al. [17] used Grey system model to predict changes of nine key parameters in an agroecosystem and energy efficiencies of the crop subsystem in the area, whereas Grey model was used to predict the manpower of undergraduate educational systems in Vietnam [18]. GM(1,1) model is the most popular Grey forecasting model, which consists of a first order differential equation with a single variable. The paper predicts inputs/outputs in the next four years through this model.

Comparing with traditional models of DEA and utilizing outputs of GM(1,1), we can discriminate efficient DMUs and rank the efficient DMUs by super-SBM and Malmquist. In short, the problem measuring productive efficiency of an industry, especially in the future, is very significant to both an economic theorist and a policy marker [19].

## 3. Methodology

### 3.1. Data Collection

The clarity of the data used in this study was collected from a market observation posting website in India. Then, the stock markets including Bombay Stock Exchange (BSE) and National Stock Exchange (NSE), which are among the famous and biggest ones in India, record their realistic financial reports. So, we have probed the required financial statements and the corresponding evaluation attributes (staff cost, energy purchase, total expenses, equity capital, net income, net profit, and EPS were taken for this study) are collected and tabled for analysis.

Even though the companies of this industrial segment are directly under the control of the Government of India, some of them have not been listed on either of the major stock exchanges like Bombay Stock Exchange (BSE) and National Stock Exchange (NSE). Therefore, this study skips those unlisted companies for our study consideration. The final selected companies taken for this research randomly of 14 companies were named (Decision Making Unit) DMU1 to DMU14 accordingly. We follow this naming scheme on these companies throughout this study while applying the DEA and Grey systems theory methods just for the sake of convenience.

Tables 1, 2, and 3 give whole picture of the real market data for all companies (DMUs) for the recent years (March 2010 to March 2014). This data will give the clear idea on initial data and its diversity in nature, and it also clearly gives a picture on the nature of the data. Moreover, according to Ittner and Larcker [20], Baier et al. [21], and Simpson and Kohers [22], seven factors, which are staff cost, energy purchase, total expenses, equity capital, net income, net profit, and EPS, are considered as the key financial indicators directly contributing to the performance of the industry. As in the tables, the data of input variables including* staff cost, energy purchase, *and* total expenses *are demonstrated in the minus values. That is natural of data collected, which means these companies use the minus digit to record the spending while running businesses.

**Figure 1:**
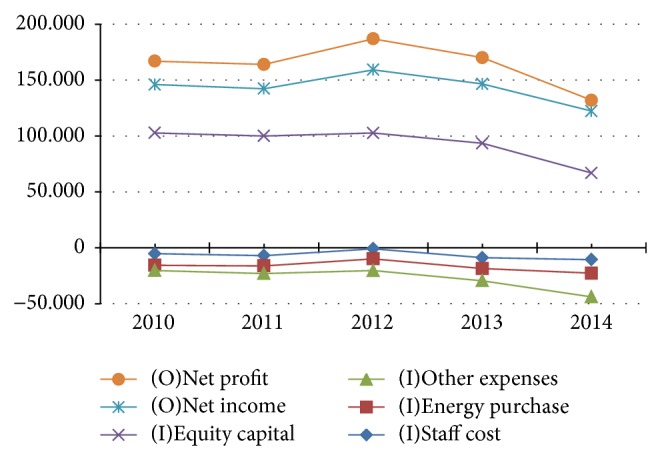
Financial results of DMU1 from 2010 to 2014, except EPS.

To have a visual description of data, we use Figure 1 as an example of the financial results of DMU1 Narmada Hydroelectric Power Corporation from 2010 to 2014. This figure drops the EPS out because all the parameters are in Indian Rs. Millions, but EPS is just calculated by Indian rupee.

### 3.2. Data Envelopment Analysis Models

The Malmquist index evaluates the efficiency change of a DMU between two time periods. It is defined as the product of “catch-up” and “frontier-shift” terms. The catch-up term is related to the degree of efforts that the DMU attained for improving its efficiency, while the frontier-shift term reflects the change in the efficient frontiers surrounding the DMU between the two time periods 1 and 2. We denote DMU_o_ at the time periods 1 and 2 by (*x*_*o*_^1^, *y*_*o*_^1^) and (*x*_*o*_^2^, *y*_*o*_^2^), respectively. We employ the following notation for the efficiency score of DMU (*x*_0_, *y*_0_)^*t*_1_^ measured by the frontier technology *t*_2_: *δ* ^*t*_2_^((*x*_0_, *y*_0_)^*t*_1_^) (*t*_1_ = 1,2 and *t*_2_ = 1,2).

Then, the catch-up effect is measured by the following formula:

(1) $$\begin{array}{c} C=\frac{\delta^{2}\left({\left(x_{0},y_{0}\right)}^{2}\right)}{\delta^{1}\left({\left(x_{0},y_{0}\right)}^{1}\right)}. \end{array}$$

The frontier-shift effect is described as

(2) $$\begin{array}{c} F={\left[\frac{\delta^{1}\left({\left(x_{0},y_{0}\right)}^{1}\right)}{\delta^{2}\left({\left(x_{0},y_{0}\right)}^{1}\right)}\times \frac{\delta^{1}\left({\left(x_{0},y_{0}\right)}^{2}\right)}{\delta^{2}\left({\left(x_{0},y_{0}\right)}^{2}\right)}\right]}^{1/2}. \end{array}$$

Malmquist index (MI) is the product of *C* and *F*; that is, Malmquist index = (catch-up) × (frontier-shift) or MI = *C*∗*F* or

(3) $$\begin{array}{c} \text{MI}={\left[\frac{\delta^{1}\left({\left(x_{0},y_{0}\right)}^{2}\right)}{\delta^{1}\left({\left(x_{0},y_{0}\right)}^{1}\right)}\times \frac{\delta^{2}\left({\left(x_{0},y_{0}\right)}^{2}\right)}{\delta^{2}\left({\left(x_{0},y_{0}\right)}^{1}\right)}\right]}^{1/2}; \end{array}$$

(*C*); (*F*); (MI) > 1 indicates progress in relative efficiency from period 1 to period 2, while (*C*); (*F*); (MI) = 1 and (*C*); (*F*); (MI) < 1 indicate the status quo and regress in efficiency, respectively.

*(Note that DEA efficiency is considered a distance measure in the literature as it reflects the efficiency of converting inputs to outputs [23].)*

We can develop the* output-oriented MI* as well by means of the* output-oriented radial* DEA models. The output-oriented models take all output slacks into account but no input slacks. This is explained below* within score in output-orientation (O-V)*

(4) δsx0,y0s=min⁡θ,λ⁡θsubject  to x0s≥Xsλllllllllllllllllll1θy0s≤YsλllllllllllllllllllL≤eλ≤Ullllllllllllllllllλ≥0.

*Intertemporal score in output-orientation (O-V)*

(5) δsx0,y0t=min⁡θ,λ⁡θ subject  to   x0t≥Xsλllllllllllllllllll1θy0t≤YsλllllllllllllllllllL≤eλ≤Ullllllllllllllllllλ≥0.

The radial approaches suffer from one general problem, that is, the neglect of slacks. In an effort to overcome this problem, Tone [24, 25] has developed the* nonradial measures of efficiency and super-efficiency: slacks-based measure (SBM) and super-SBM*. Using these measures we develop here the nonradial and slacks-based MI. In the* output-oriented* case, we solve the following LPs.

*SBM-O*

(6) δtx0,y0s=min⁡λ,s+⁡11+((1/q)∑i=1qsi+/yi0s)   subject  to x0s≥Xtλllllllllllllllllly0s=Ytλ−s+lllllllllllllllllL≤eλ≤Ulllllllllllllllllλ≥0, s+≥0,

where the vector *s* ^+^ ∈ *R* ^*q*^ denotes the output-slacks.

*Super-SBM-O*

(7) δtx0,y0s=min⁡λ,s+⁡11−((1/q)∑i=1qsi+/yi0t) subject  to   x0s≥Xtλlllllllllllllllllllly0s≤Ytλ+s+llllllllllllllllllllL≤eλ≤Ullllllllllllllllllllλ≥0, s+≥0.

### 3.3. GM(1,1) Model: Forecasting Process

The researchers use GM(1,1) model to predict the realistic input/output factors for the next 4 years (2015 to 2018). Following, the study takes company DMU2 as an example to understand how to compute in GM(1,1) model in period 2010–2014, specifically* net income of* DMU2 as an example to explain for calculation procedure, and other variables are calculated in the same way. The procedure is carried out step by step as follows.

First, the researchers use the GM(1,1) model for trying to forecast the variance of primitive series as follows.

 First, create the primitive series:
  (8) X(0)=482,213.2;583,597.8;620,535.8;llllll656,739.3;720,189.3.
 Second, perform the accumulated generating operation (AGO):
  (9) X(1)=482,213.2;1,065,811.00;1,686,346.80;lllllll2,343,086.10;3,063,275.40,x11=x0(1)=482,213.2,x12=x01+x02=1,065,811.00,x13=x(0)(1)+x(0)(2)+x(0)(3)=1,686,346.80,x14=x(0)(1)+x(0)(2)+x(0)(3)+x(0)(4)=2,343,086.10,x15=x(0)(1)+x(0)(2)+x(0)(3)+x(0)(4)+x(0)(5)=3,063,275.40.
 Third, create the different equations of GM(1, 1).

To find *X* ^(1)^ series, and the following mean obtained by the mean equation is

(10) $$\begin{array}{c} z^{\left(1\right)}\left(2\right)=\frac{1}{2}\left(\text{482,213.2}+\text{1,065,811.00}\right)=\text{774,012.1},\\ z^{\left(1\right)}\left(3\right)=\frac{1}{2}\left(\text{1,065,811.00}+\text{1,686,346.80}\right)=\text{1,376,078.90},\\ z^{\left(1\right)}\left(4\right)=\frac{1}{2}\left(\text{1,686,346.80}+\text{2,343,086.10}\right)=\text{2,014,716.45},\\ z^{\left(1\right)}\left(5\right)=\frac{1}{2}\left(\text{2,343,086.10}+\text{3,063,275.40}\right)=\text{2,703,180.75}. \end{array}$$

 Fourth, solve equations.

To find *a* and *b*, the primitive series values are substituted into the Grey differential equation to obtain

(11) $$\begin{array}{c} \text{583,597.8}+a\times \text{774,012.1}=b,\\ \text{620,535.8}+a\times \text{1,376,078.90}=b,\\ \text{656,739.3}+a\times \text{2,014,716.45}=b,\\ \text{720,189.3}+a\times \text{2,703,180.75}=b. \end{array}$$

Convert the linear equations into the form of a matrix.

Let

(12) $$\begin{array}{c} B=\left[\begin{array}{cc} -\text{774,012.1} & 1\\ -\text{1,376,078.9} & 1\\ -\text{2,014,716.45} & 1\\ -\text{2,703,180.75} & 1 \end{array}\right],\;\;\hat{\theta }=\left[\begin{array}{c} a\\ b \end{array}\right],\\ y_{N}=\left[\begin{array}{c} \text{583,797.8}\\ \text{620,535.8}\\ \text{656,739.3}\\ \text{720,189.3} \end{array}\right]. \end{array}$$

And then use the least square method to find *a* and *b*:

(13) $$\begin{array}{c} \left[\begin{array}{c} a\\ b \end{array}\right]=\hat{\theta }={\left(B^{T}B\right)}^{-1}B^{T}y_{N}=\left[\begin{array}{c} -0.0696\\ 525721.919 \end{array}\right]. \end{array}$$

Use the two coefficients *a* and *b* to generate the whitening equation of the differential equation:

(14) $$\begin{array}{c} \frac{dx^{\left(1\right)}}{dt}-0.0696\times x^{\left(1\right)}=\text{525721.919}. \end{array}$$

Find the prediction model from

(15) X1k+1=X01−bae−ak+ba,x1k+1=482213.2−525721.919−0.0696e0.0696k +525721.919−0.0696=(8035689.05)e0.0696k−7553475.852.

Substitute different values of *k* into the equation:

(16) $$\begin{array}{c} k=0\;X^{\left(1\right)}\left(1\right)=\text{482,213.20},\\ k=1\;X^{\left(1\right)}\left(2\right)=\text{1,065,811.00},\\ k=2\;X^{\left(1\right)}\left(3\right)=\text{1,204,133.60},\\ k=3\;X^{\left(1\right)}\left(4\right)=\text{1,277,275.10},\\ k=4\;X^{\left(1\right)}\left(5\right)=\text{1,376,928.60},\\ k=5\;X^{\left(1\right)}\left(6\right)=\text{1,485,426.76},\\ k=6\;X^{\left(1\right)}\left(7\right)=\text{1,585,652.09},\\ k=7\;X^{\left(1\right)}\left(8\right)=\text{1,699,984.96},\\ k=8\;X^{\left(1\right)}\left(9\right)=\text{1,822,561.76}. \end{array}$$

Derive the predicted value of the original series according to the accumulated generating operation and obtain

(17) x^01=x1(1)=482,213.20—for  the  year  2010,x^02=x12−x11=583,597.80—forecasted  for  2011,x^03=x13−x12=620,535.80—forecasted  for  2012,x^04=x14−x13=656,739.30—forecasted  for  2013,x^05=x15−x14=720,189.30—forecasted  for  2014,x^06=x16−x15=765,237.46—forecasted  for  2015,x^07=x17−x16=820,414.63—forecasted  for  2016,x^08=x18−x17=879,570.33—forecasted  for  2017,x^09=x19−x18=942,991.43—forecasted  for  2018.

Similarly to the above computation process, the study could get the forecasting results of all DMUs from 2015 and 2018; the detailed numbers are shown in Table 4, respectively.

*Forecasting Accuracy*. It is undeniable that forecasting always has some errors; they are essentially about prediction of the future in uncompleted information. Thus, in this paper, the MAPE (mean absolute percent error) is employed to measure the accuracy of a method for constructing fitted time series values in statistics. MAPE is often used to measure forecasting accuracy. In the book of Stevenson [26], it is stated clearly that MAPE is the average absolute percent error which measures accuracy in a fitted time series value in statistics, specifically trending. Consider MAPE = (1/*n*)∑(|Actual − Forecast|/Actual) × 100; *n* is forecasting number of steps.

The parameters of MAPE show the forecasting ability as follows:

 MAPE < 10% “Excellent,”
 10% < MAPE < 20% “Good,”
 20% < MAPE < 50% “Reasonable,”
 MAPE > 50% “Poor.”

### 3.4. DEA-GM Model for Performance Measurement

**Figure 2:**
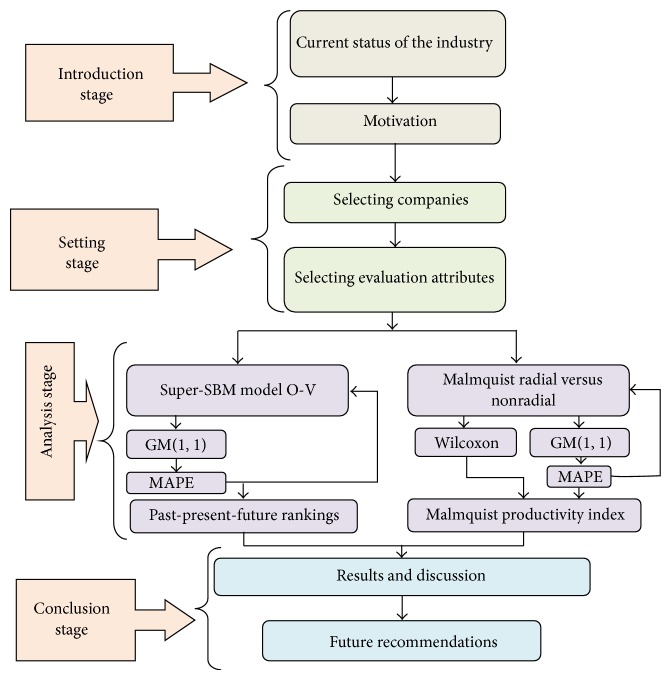
Research process.

The original DEA used past data to evaluate the past performances. And then it is said that the future performances could be similar to the past ones. The paper uses GM by past data to forecast the future data and then uses the future data for inputting DEA to evaluate the future performances. In this way, the trend of each DMU can be considered much better than original DEA. Moreover, the primary objective of this model is to overcome the ranking inefficiency and to eliminate the subjective evaluation of DEA. According to the method, the judging matrix is formed by using the outputs of GM(1,1) as inputs for DEA models. This method consists of the following steps (Figure 2).

The setting stage is mentioned early, which is about introduction, motivation, selecting companies, and selecting attributes of these firms. After the setting stage, we go to the analysis stage at which research models are applied. In performing evaluation by ranking, Super-SBM is employed. GM(1,1) is used to forecast the parameters that then can be used for future estimated ranking among electricity companies. On the other side,* Malmquist nonradial and radial* models are applied to demonstrate performance evaluation. However, we need to see whether significant differences exist between these models and then Wilcoxon can handle this task. Again, the results of GM(1,1), which were tested for the accuracy by mean absolute percent error (MAPE), are utilized to see future trends. Finally, we could easily analyze the efficiency change, technical change, and productivity index based MPI.

## 4. Results and Future Analysis

### 4.1. Forecasting Results

Similarly to the above computation process (mentioned in Section 3.3), the study could get the forecasting results of all DMUs from 2015 and 2018. Due to the large size of forecasting results, the detailed numbers of outputs are shown as a typical example in Table 4.

Moreover, the forecasting accuracy is very important to solve the mathematical concerns about the forecasting method, so the results of MAPE are listed in Table 5. The calculations of MAPE are almost smaller than 10%, especially the average MAPE of 14 DMUs which reaches 9.24% (below 10% as well) and it strongly confirms that the GM(1,1) model provides highly accurate prediction. Moreover, if the MAPE is over 10% due to our strict accuracy, it has to be rechecked and reselected as shown in Figure 1.

### 4.2. Pearson Correlation

To apply DEA model, we have to make sure the relationship between input and output factors is isotonicity, which means that if the input quantity increases the output quantity could not decrease under the same condition [27]. Firstly, we conducted a simple correlation test* Pearson correlation* to measure the degree of association between two variables. Higher correlation coefficient means closer relation between two variables while lower correlation coefficient means that they are less correlated.

The interpretation of the correlation coefficient is explained in more detail as follows. The correlation coefficient is always between −1 and +1. The closer the correlation is to +/−1, the closer it is to a perfect linear relationship. Its general meaning was shown in Table 6.

In the empirical study, the results in Table 7 indicate that the correlation complies well with the prerequisite condition of the DEA model because their correlation coefficient shows strong positive associations. Therefore, these positive correlations also demonstrate very clearly the fact that the researcher's choice of input and output variables at the beginning is appropriate. Obviously, none of the variables' removal is necessary.

Table 7 indicates that both input and output variables are positively correlated even with minor correlation existing between EPS and other elements indicated as less than 0.2. This is easy to understand that EPS (earning per share) is calculated to be value of each share, so they cannot be as much as other factors which are summarized by raw data. From these results, we can justify the reason why we use these indicators for DEA methodologies. The correlation is also very significant which will affect the performance.

### 4.3. Performance Rankings: Super-SBM

Table 8 summarizes the analysis process of March, 2014 data. They are set at value* Returns to Scale = Variable (Sum of Lambda = 1)*.

Table 8 indicates that the number of inefficient DMUs is 8 at March 2014. This clearly indicates that super-SBM can distinguish all DMUs with significant differences on their scoring. Then, the results reflect that a large number of inefficient electricity companies still exist, and we will go to a deeper analysis in the next section.

Table 9 shows the consolidated DEA super-SBM efficiency scores for the last-5-year data and rankings of DMUs by their scores. This indicates that the ranking of the industries is tending to change in a very slight manner on yearly basis. However, the majority of these companies are maintaining their “efficient” levels even after yearly changes on their financial nature.

From Table 10, we used the forecasted results by applying GM(1,1) to come up with these rankings. In the future, obviously these electricity companies are keeping their performance over their partners. There are just light changes between the efficiency scores. However, we can still see some of the companies are under “1” of efficiency-inefficiency.

### 4.4. Performance Efficiency Evaluation: Malmquist Radial versus Malmquist Nonradial

Boles et al. [28] stated that the performance efficiency evaluation is very important to see whether the industry is on the progress in development or not. Thus, the researchers in this case have to be very careful in choosing models to make sure of the accuracy of the performance evaluation process. Firstly, we used the two models:* Malmquist radial and Malmquist nonradial* (mentioned in Section 3.2). Then, we get the results of Malmquist (see Table 11). Malmquist radial has the average score of 1.011246 compared with 1.402955 of Malmquist nonradial (SD = 0.126337 and 1.10269 of these two models, resp.).

However, it is very difficult to see whether they have significant differences just by comparing some statistical descriptions. Thus, we used Wilcoxon to test the differences. We, then, set up the null hypothesis “*There is no difference of performance efficiency evaluation between Malmquist radial and Malmquist nonradial.*”

Table 12 shows the correlations between two paired samples at *n* = 14, correlation = 0.586, *P* = 0.028, *P* < 0.05, which means that there is significant difference between correlation of two models mentioned.

Next, we come up with the results of Wilcoxon test (Table 13), which are *M* = 0.39, SD = 1.03, 95%  CI = −0.205; 0.989, *t* = 1.42, *df* = 13, and *P* = 0.18, in which 95%confidence interval of the difference goes through 0 and *P*value >0.05. That means that we accept the null hypothesis; that is, there is no significant difference between the* Malmquist radial and Malmquist nonradial* to test the performance evaluation. However, this study will use one type of Malmquist which is nonradial O-V, as it is mentioned above that the radial approaches suffer from one general problem, that is, the neglect of slacks. In an effort to overcome this problem, Tone [24, 25] has developed the nonradial measures of efficiency and super-efficiency.

**Figure 3:**
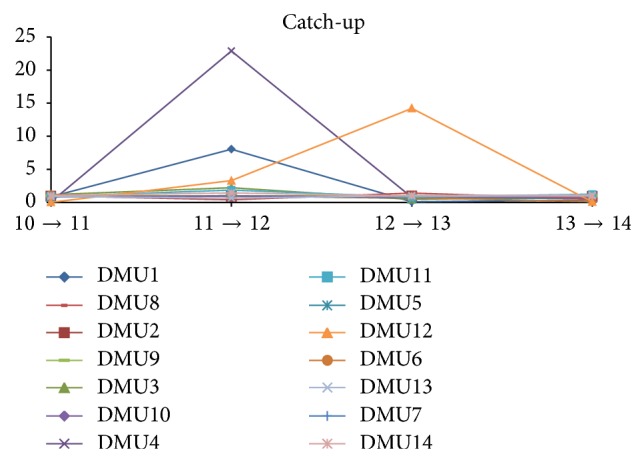
Efficiency (catch-up) change over the period from March 2010 to March 2014.

Table 14 and Figure 3 show the efficiency change or what is named “catch-up” of the India electricity industry over the yearly periods of time interval. This reveals that the efficiency changes are not so consistent due to the fact that their nature of their financial management is not really consistently improving or is not consistently outperforming DMU over the time yearly frame. They figure out that the “efficiency changes” among the companies over the yearly time frame are exhibiting the inconsistency over the years.

Nevertheless, we can notice some wild fluctuations of the changes which are among DMU1* (NHPC)*, DMU4* (REC),* and DMU12* (Reliance)*, and the rest of them have no such big changes even with slight changes by slight increases and decreases around the catch-up indices of 0–2. This clearly indicates that the entire industry has not shown much big changes on their catch-up scores during the last 4 years. It is owing to the fact that the financial segment is not much affected even after the global economic recession in 2007-2008 [29].

**Figure 4:**
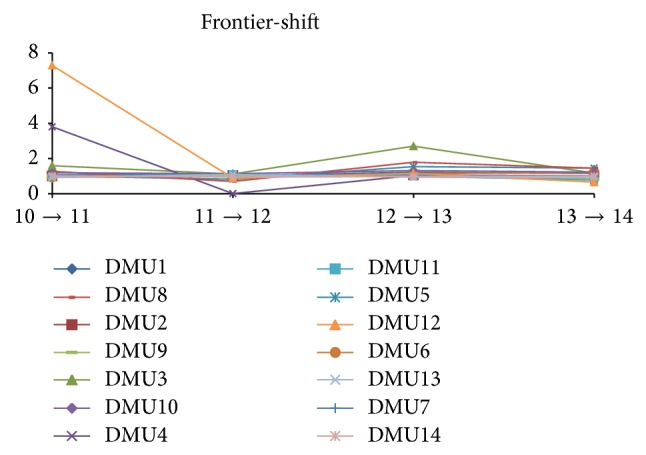
Technical (frontier) change over the period from March 2010 to March 2014.

The technical or the frontier-shift changes of the companies in the Indian electricity industry are shown in Table 15 and Figure 4. At the first sight, we noticed that the companies are tending to change their level of technical changes or their innovation effect inconsistently. This is almost like the same effect in the previous “efficiency change” level.

However, as mentioned early, the DMU4* (REC)* and DMU12* (Reliance Power)* have their up-and-down changes in efficiency, which again notably made some abruptness in technical changes over the beginning years and then went smoothly with the overall trend of the companies in the industry.

**Figure 5:**
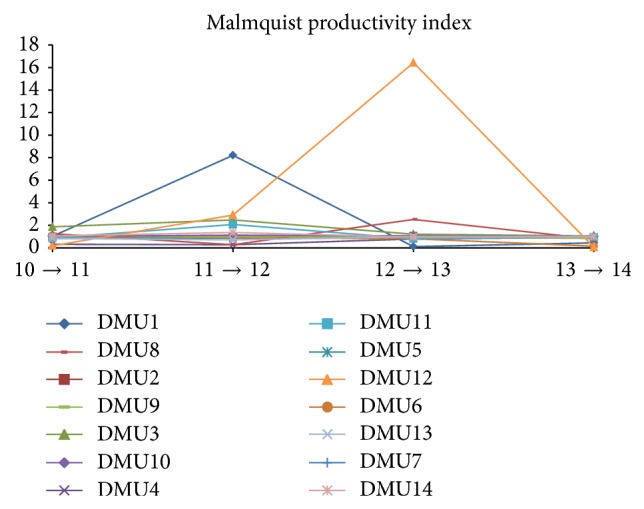
Productivity index (MPI) change over the period from March 2010 to March 2014.

Finally, the most important element in the performance evaluation of the industry is Malmquist productivity index (MPI), which is clearly indicated in Table 16 and Figure 5. Overall, most of the companies have done well in their performance when the indices are larger than 1 (>1).

Notably, DMU4 and DMU12's MPIs were shaking over the period, and finally in March 2014 they got close to 0. The rest of the companies have also increased their MPI scores but very slightly. And the DMU1, DMU3, DMU7, DMU8, DMU10, DMU11, and DMU14 have shown a decrement of their productivity index scores.

**Figure 6:**
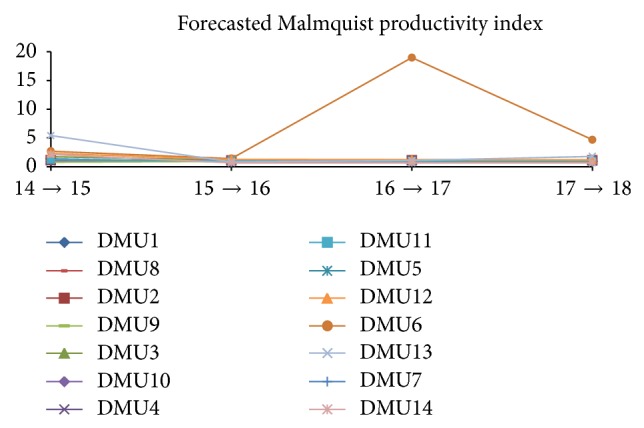
MPI change over the forecasted period from 2014 to 2018.

For the future of the industry, GM(1,1) handled the task of forecasting financial performance of the companies (input and output variables). As mentioned previously, MPI is done by Malmquist nonradial O-V model, which is illustrated in Table 17 and Figure 6. Obviously, we can see the stable changes of the industry in the forecasting period (2014–2018) in which almost MPIs of companies reach the “efficiency” level or positive change year over year.

Moreover, we noticed the consistent change of DMU7* (CESC).* Through this index, 3 consecutive years at over 1.05 of efficiency level, we would consolidate the accuracy in forecasting of GM(1,1).

In the future, we also mention DMU6 as a new element of the industry when its MPI rockets up to the level of over 18; then in the whole period it keeps going up at around 5. In contrast, DMU14 will not perform well according to the forecasting results; apparently, as shown in Table 17 it only reaches efficiency over 2014-2015 and then keeps inefficient performance over the next few years (at 0.65).

## 5. Discussions

In this study, 14 qualified companies in the Indian electricity industry are involved, and the results of rankings from of super-SBM model show the order of performance scores on the top DMU4, DMU2, DMU11, DMU13, DMU7, and DMU9. Moreover, these companies still keep their top according to forecasting results except some changes. In the period 2015–2018, DMU4, DMU2, and DMU11 are in the top 3 positions; DMU7 and DMU9 move to positions 4 and 5; DMU3 comes at position 6, when DMU13 moves to position 9 in the board (see Table 18).

In lowest score, we notice that DMU1 and DMU12 are in the last 4 positions over the past-present-future period. They need a very serious improvement action if they want to take over competitors or partners in the industry since super-SBM model can distinguish the rankings of all DMUs and we would derive a clear decision for improving the performance of less efficiency scored DMUs.

After applying two Malmquist models and selecting out one suitable MPI methodology to our data set and calculating the efficiency scores, we have found that all companies in the industry have not shown many abrupt changes on their scores and it is always not consistently good or is consistently standing out. Thus, it means that there are not many changes happening on Indian stock markets even with financial crisis in a broad context, except some firms which are previously analyzed. This study provides many significant and noticeable results after applying each methodology for making necessary decisions on the respective concerns. This completed and integrated numerical study gives us better insights through the integration method as it minimizes the methodology limitation problems.

## 6. Conclusions

It gives better insights in terms of understanding the impact of global economic recession and its consequences in India as it is a core industry of the economy. This also makes this research a considerable study for global economic outlook planners and researchers. Furthermore, then according to forecasted MPI, companies with inefficient level (<1) need to be positive in changing or improving their management activities, business trends or size, or any other methods to make progress in the future time.

In our limitations of this research, researchers would like to contribute to the overview about the Indian electricity industry and accordingly implemented the integrated research methodologies to give out meaningful and helpful results to the development of the industry. We would also suggest that this study could be considered to be a better model of performance analysis among the decision makers of variety of industries. However, the completely integrated performance analysis model needs a detailed reevaluation in terms of the type of industry that it can be applied to, the nature of the input data, adoptability of data for each method, applicability of each method, and so on. The future directions can also be suggested for including or avoiding different methodologies in this completely integrated model.

## Figures and Tables

**Table 1:** Financial results of electricity companies in March 2010.

DMUs	Inputs (Rs. millions)				Outputs (Rs. millions; except EPS)		
	(I) Staff cost	(I) Energy purchase	(I) Other expenses	(I) Equity capital	(O) Net income	(O) Net profit	Basic EPS
Narmada Hydroelectric Power Corporation							
DMU1	−5298.4	−10332.5	−4698.4	123,007.4	43,319.80	28,591.60	1.76
NTPC Electric Supply Company							
DMU2	−24123.6	−294627.4	−46771.6	82,454.60	482,213.20	126,943.9	10.59
Power Grid Corporation of India							
DMU3	−7267.0	−19796.9	−5313.6	42,088.40	71,274.50	42,658.30	4.85
REC Power Distribution Company							
DMU4	−1171.0	−23.8	−277.8	9,874.60	65,497.60	65,603.40	23.06
Nathpa Jhakri Dam (SJVN)							
DMU5	−745.1	−4332.8	−2105.4	41,088.10	17,697.40	11,904.00	2.37
Adani Power							
DMU6	−45.5	−1667.1	−543.5	21,800.35	4,348.61	2,411.73	0.82
CESC Limited							
DMU7	−3790.0	−17140.0	−6560.0	1,260.00	33,650.00	7,000.00	34.68
JSW Energy							
DMU8	−605.0	−9268.4	−2219.8	16,400.50	23,728.70	12,317.10	5.86
KSK Energy Ventures							
DMU9	−77.2	−13.8	−186.1	3,726.30	2,145.01	2,481.53	3.57
Neyveli Lignite Corporation							
DMU10	−16965.3	−5014.7	−8864.9	16,777.10	41,210.30	16,384.40	7.44
Reliance Infrastructure							
DMU11	−6528.6	−75784.5	−9966.3	2,449.10	100,272.60	15,891.50	51.11
Reliance Power							
DMU12	−389.3	−467.8	−134.3	23,968.00	85.51	2,889.38	1.14
Tata Power							
DMU13	−3052.9	−43394.0	−10692.6	2,373.30	70,982.70	16,659.00	40.77
Torrent Power							
DMU14	−2181.2	−32817.9	−9557.4	4,724.50	59,092.00	15,008.20	17.71

**Table 2:** Financial results of electricity companies in March 2011 and March 2012.

DMUs	Inputs (Rs. millions)								Outputs (Rs. millions; except EPS)					
	(I) Staff cost		(I) Energy purchase		(I) Other expenses		(I) Equity capital		(O) Net income		(O) Net profit		(O) Basic EPS	
	2011	2012	2011	2012	2011	2012	2011	2012	2011	2012	2011	2012	2011	2012
DMU1	−6996.2	−906.8	−9167.4	−8927.4	−6801.4	−10543.7	123,007.4	123,007.4	42,252.5	56,546.9	32,919.9	37,904.2	1.76	2.25
DMU2	−27897.1	−30904.8	−353737.8	−416354.6	−68856.7	−60682.6	82,454.6	82,454.6	583,597.8	620,535.8	141,986.8	140,378.0	11.04	11.19
DMU3	−7458.9	−8429.7	−21993.9	−25725.4	−5914.9	−8099.8	46,297.3	46,297.3	83,887.0	100,353.3	55,630.3	65,595.2	6.19	7.03
DMU4	−1274.7	−1709.7	−32.6	−555.4	−369.0	−1109.0	9,874.6	9,874.6	82,569.1	103,375.9	83,276.3	101,716.6	26.03	28.53
DMU5	−847.2	−1111.5	−4442.1	−4460.0	−1169.0	−1373.7	41,366.3	41,366.3	18,126.7	19,275.0	13,145.7	14,422.7	2.21	2.58
DMU6	−297.7	−525.1	−7043.6	−22644.9	−3368.9	−9439.2	21,800.4	21,800.4	21,064.3	39,489.0	10,540.5	9,789.1	2.40	−1.35
DMU7	−4330.0	−4710.0	−20930.0	−23980.0	−6800.0	−9440.0	1,260.0	1,260.0	40,180.0	46,810.0	8,860.0	9,690.0	39.09	44.37
DMU8	−669.3	−809.0	−20780.5	−34602.8	−4057.1	−6382.4	16,400.5	16,400.5	38,619.3	50,164.2	14,304.6	9,510.2	5.40	1.43
DMU9	−109.9	−99.4	−81.3	−32.6	−97.7	−115.9	3,726.3	3,726.3	1,596.8	675.2	1,344.0	1,478.7	0.77	0.00
DMU10	−14007.9	−16982.0	−5109.3	−5585.3	−11640.9	−13032.0	16,777.1	16,777.1	39,490.8	48,668.5	18,436.2	20,552.8	7.74	8.41
DMU11	−7621.8	−7404.8	−69969.6	−137455.3	−9911.4	−10135.4	2,674.7	2,630.3	96,145.9	179,066.7	13,775.0	30,658.6	43.23	75.70
DMU12	−606.0	−386.6	−671.6	−277.9	−477.5	−941.4	28,051.3	28,051.3	363.8	661.2	2,960.1	3,712.7	1.06	1.11
DMU13	−3411.2	−5126.5	−43471.0	−54391.9	−11946.0	−13297.2	2,373.3	2,373.3	69,184.8	84,958.4	15,292.4	21,977.4	40.84	4.53
DMU14	−2731.2	−2415.9	−38233.6	−47099.8	−10628.2	−10781.5	4,724.5	4,724.5	68,345.6	79,178.2	17,677.2	19,899.4	22.56	26.19

**Table 3:** Financial results of electricity companies in March 2013 and March 2014.

DMUs	Inputs (Rs. millions)								Outputs (Rs. millions; except EPS)					
	(I) Staff cost		(I) Energy purchase		(I) Other expenses		(I) Equity capital		(O) Net income		(O) Net profit		(O) Basic EPS	
	2013	2014	2013	2014	2013	2014	2013	2014	2013	2014	2013	2014	2013	2014
DMU1	−8,874.1	−10,586.7	−9,692.9	−12,107.6	−10,953.3	−21,191.0	123007.40	110,706.70	53066.40	55,370.40	33475.00	9,787.90	1.91	0.82
DMU2	−33,601.2	−38,679.9	−410,182.5	−458,297.1	−75,782.6	−86,988.8	82454.60	82,454.60	656739.30	720,189.30	168188.80	109,747.40	15.30	13.31
DMU3	−8,864.0	−9,416.8	−33,519.2	−39,956.8	−9,350.4	−13,527.8	46297.30	52,315.90	127578.50	152,302.80	81553.80	44,974.20	9.15	9.36
DMU4	−1,518.4	−1,299.1	−1,344.3	−3,162.3	−1,422.0	−1,050.8	9874.60	9,874.60	135188.60	170,179.80	131702.00	46,837.00	38.66	47.43
DMU5	−1,095.4	−1,237.5	−4,466.7	−4,745.2	−1,217.7	−1,453.8	41366.30	41,366.30	16821.00	18,735.80	12386.40	11,146.30	2.54	2.69
DMU6	−1,373.9	−1,651.7	−46,988.8	−61,557.8	−18,379.4	−18,836.5	23932.70	28,719.20	63329.80	110,100.40	1938.80	5,952.60	−8.16	2.13
DMU7	−5,590.0	−6,940.0	−27,420.0	−18,610.0	−9,980.0	−18,610.0	1260.00	1,260.00	53170.00	55,100.00	11110.00	6,520.00	49.50	52.18
DMU8	−1,065.0	−889.7	−38,942.8	−36,427.7	−2,932.4	−2,469.1	16400.50	16,400.50	63964.50	58,026.10	19855.80	6,024.80	6.05	3.67
DMU9	−64.1	−55.0	−16.6	−10.5	−101.6	−59.6	3726.30	3,726.30	491.55	479.76	1295.42	97.50	0.11	0.01
DMU10	−19,524.2	−21,945.9	−6,038.3	−7,005.4	−15,370.7	−16,331.8	16777.10	16,777.10	55900.70	59,672.30	20797.00	15,018.80	8.70	8.95
DMU11	−8,561.3	−8,231.1	−107,261.2	−78,600.4	−12,160.7	−11,051.0	2630.30	2,625.80	143220.30	113,569.30	26042.70	15,879.40	76.03	60.38
DMU12	−476.3	−412.5	−94.3	−168.4	−656.0	−840.1	28051.30	27,966.30	120.10	916.90	1281.90	564.80	1.83	0.20
DMU13	−5,476.0	−5,449.5	−60,185.4	−47,878.3	−13,135.6	−13,271.1	2373.30	2,373.30	95672.80	86,270.40	23816.30	9,540.80	3.44	3.50
DMU14	−2,490.0	−2,703.3	−57,987.4	−62,745.8	−11,910.1	−13,489.3	4724.50	4,724.50	81298.70	85,756.20	10312.20	948.40	8.15	2.01

**Table 4:** Forecasted values of outputs of all DMUs from 2015 to 2018.

DMUs	Outputs (Rs. millions, except EPS)											
	(O) Net income				(O) Net profit				(O) EPS			
	′15	′16	′17	′18	′15	′16	′17	′18	′15	′16	′17	′18
DMU1	60,946.31	65,111.53	69,561.41	74,315.41	13,700.67	11,681.34	9,959.64	8,491.70	1.13	0.97	0.83	0.71
DMU2	765,237.46	820,414.63	879,570.33	942,991.43	128,350.01	139,444.74	151,498.51	164,594.23	15.56	16.91	18.37	19.95
DMU3	186,345.07	227,943.82	278,828.86	341,073.23	55,032.05	65,220.98	77,296.36	91,607.43	11.20	12.92	14.92	17.22
DMU4	214,523.71	272,864.48	347,071.31	441,459.05	57,571.58	71,518.24	88,843.47	110,365.71	58.30	72.41	89.95	111.73
DMU5	18,362.81	18,440.79	18,519.11	18,597.75	11,900.86	12,585.87	13,310.31	14,076.44	2.87	3.03	3.20	3.38
DMU6	207,817.86	362,130.80	631,027.17	1,099,589.67	5,752.60	6,952.60	5,952.60	5,354.60	1.80	2.02	2.28	2.56
DMU7	62,616.77	69,374.97	76,862.57	85,158.31	7,282.40	8,007.79	8,805.43	9,682.53	58.28	64.06	70.43	77.42
DMU8	71,750.08	81,500.12	92,575.08	105,155.01	6,548.38	6,454.80	6,362.57	6,271.65	3.99	3.93	3.87	3.81
DMU9	177.81	104.72	61.68	36.33	21.79	11.57	6.15	3.26	0.11	0.01	0.00	0.77
DMU10	69,654.52	79,310.72	90,305.56	102,824.62	15,882.79	16,630.55	17,413.51	18,233.34	9.46	9.91	10.37	10.86
DMU11	136,523.48	137,961.56	139,414.80	140,883.34	20,204.24	21,843.81	23,616.42	25,532.88	75.86	81.36	87.26	93.59
DMU12	923.39	1,203.80	1,569.36	2,045.93	2,168.34	1,936.13	1,728.78	1,543.64	0.75	0.66	0.58	0.51
DMU13	99,643.75	106,807.09	114,485.40	122,715.70	9,846.78	9,673.31	9,502.90	9,335.48	36.90	9.72	2.56	0.67
DMU14	92,908.45	99,441.17	106,433.22	113,916.92	2,094.62	1,346.20	865.20	556.06	4.43	2.85	1.83	1.18

Source: calculated by researchers.

**Table 5:** Average MAPE of DMUs.

DMUs	Average MAPE	DMUs	Average MAPE
DMU1	12.03%	DMU8	16.34%
DMU2	3.13%	DMU9	20.17%
DMU3	1.71%	DMU10	1.24%
DMU4	3.31%	DMU11	13.70%
DMU5	1.98%	DMU12	24.00%
DMU6	9.89%	DMU13	5.63%
DMU7	1.71%	DMU14	14.45%

Average of all MAPEs 9.24%.			

**Table 6:** The Pearson correlation coefficient.

Correlation coefficient	Degree of correlation
>0.8	Very high
0.6–0.8	High
0.4–0.6	Medium
0.2–0.4	Low
<0.2	Very low

**Table 7:** Correlation coefficient: March 2014 data.

	Staff cost	Energy purchase	Other expenses	Equity capital	Net income	Net profit	Basic EPS
Staff cost	1	0.8094686	0.8975678	0.4946254	0.8205856	0.779173	0.049587
Energy purchase	0.8094686	1	0.9465775	0.408433	0.9657305	0.8404919	0.0218223
Other expenses	0.8975678	0.9465775	1	0.5341933	0.9271514	0.8063402	0.0046019
Equity capital	0.4946254	0.408433	0.5341933	1	0.434463	0.4620127	−0.3408736
Net income	0.8205856	0.9657305	0.9271514	0.434463	1	0.9435955	0.1093055
Net profit	0.779173	0.8404919	0.8063402	0.4620127	0.9435955	1	0.1777289
Basic EPS	0.049587	0.0218223	0.0046019	−0.3408736	0.1093055	0.1777289	1

**Table 8:** Summary of super-SBM results.

Number of DMUs in data: 14	
Number of DMUs with inappropriate data: 0	
Number of evaluated DMUs: 14	
Average of scores: 1.9075947	
Number of efficient DMUs: 6	
Number of inefficient DMUs: 8	
Number of over iteration DMUs: 0	

**Table 9:** Past-present period scores and rankings of Indian electricity companies.

DMUs	March 2010		March 2011		March 2012		March 2013		March 2014	
	Score	Rank	Score	Rank	Score	Rank	Score	Rank	Score	Rank
DMU1	0.1805557	14	0.153255	13	1.233397	4	0.115798	13	0.044725	14
DMU2	2.1795041	2	2.089054	1	1.981437	3	1.966767	2	1.803893	3
DMU3	0.3865749	12	0.452200	10	1.005157	8	0.450591	9	0.408009	8
DMU4	11.825946	1	1.000000	5	22.898276	1	17.639098	1	17.285690	1
DMU5	0.2957943	13	0.243983	12	0.250182	13	0.157151	12	0.102082	12
DMU6	1	5	1.180909	4	1.083435	5	0.003309	14	0.094637	13
DMU7	1	5	1.000000	5	1.000000	9	1.000000	7	1.000000	5
DMU8	0.6235547	10	0.618462	9	0.266180	12	0.352507	10	0.189113	10
DMU9	1	5	1.000000	5	0.998892	10	1.000000	7	1.000000	5
DMU10	0.457855	11	0.391654	11	0.386875	11	0.311759	11	0.264296	9
DMU11	1.3396894	4	1.211566	3	2.270265	2	1.813421	3	2.235738	2
DMU12	0.9980466	8	0.025995	14	0.085796	14	1.220087	4	0.115184	11
DMU13	1.7179445	3	1.547032	2	1.011640	7	1.072382	5	1.164481	4
DMU14	0.7090125	9	0.741591	8	1.068094	6	1.048365	6	0.998477	7

**Table 10:** Future scores and rankings of Indian electricity companies.

DMUs	2015		2016		2017		2018	
	Score	Rank	Score	Rank	Score	Rank	Score	Rank
DMU1	0.046732	13	0.034523	14	0.024455	14	0.016971	14
DMU2	1.735220	2	1.625600	2	1.514369	2	1.373412	2
DMU3	0.398272	7	0.379911	6	0.356149	7	0.333810	7
DMU4	12.475861	1	11.638251	1	10.762886	1	9.799443	1
DMU5	0.082926	11	0.091276	9	0.100177	10	0.109193	10
DMU6	0.004596	14	0.067917	12	1.105600	4	1.190524	3
DMU7	1.000000	4	1.000000	4	1.000000	5	1.000000	5
DMU8	0.111457	9	0.090732	10	0.073254	12	0.058714	11
DMU9	0.999807	5	0.999595	5	0.998631	6	1.000000	5
DMU10	0.223492	8	0.194331	8	0.168504	8	0.145396	8
DMU11	1.376886	3	1.265668	3	1.147562	3	1.039725	4
DMU12	0.063641	12	0.086474	11	0.113469	9	0.134560	9
DMU13	0.679931	6	0.292033	7	0.088556	11	0.022830	12
DMU14	0.092551	10	0.053271	13	0.030293	13	0.017040	13

**Table 11:** The average indices of Malmquist radial and Malmquist nonradial models.

DMUs	Average of Malmquist radial	Average of Malmquist nonradial
DMU1	0.978992	2.452093
DMU2	1.016916	0.984933
DMU3	1.192086	1.654519
DMU4	0.75047	0.58361
DMU5	0.910147	0.898432
DMU6	1.059766	0.74262
DMU7	1.05946	1.050272
DMU8	1.089834	1.211586
DMU9	0.861731	0.950941
DMU10	1.065796	1.059666
DMU11	1.02667	1.176019
DMU12	1.226233	4.897249
DMU13	0.89831	0.888796
DMU14	1.021026	1.090631

Mean	**1.011246**	**1.402955**

Max	1.226233	4.897249

Min	0.75047	0.58361

SD	0.126337	1.102699

**Table 12:** Paired samples correlations.

	*N*	Correlation	Sig.
Nonradial and radial	14	0.586	0.028

**Table 13:** Paired samples test.

	Paired differences					*t*	df	Sig. (2-tailed)
	Mean	Std. deviation	Std. error mean	95% confidence interval of the difference			
				Lower	Upper		
Nonradial-radial	0.39171	1.03373	0.27628	−0.20515	0.98857	1.418	13	0.180

**Table 14:** Efficiency (catch-up) change over the period from March 2010 to March 2014.

Catch-up	March 10 → March 11	March 11 → March 12	March 12 → March 13	March 13 → March 14	Average
DMU1	0.848794	8.048023	0.093885	0.386234	2.344234
DMU2	0.9585	0.948485	0.992596	0.917187	0.954192
DMU3	1.16976	2.222816	0.44828	0.905497	1.186588
DMU4	0.08456	22.89828	0.770324	0.979965	6.183281
DMU5	0.824839	1.025408	0.628149	0.649576	0.781993
DMU6	1.180909	0.917459	0.705769	0.123764	0.731975
DMU7	1	1	1	1	1
DMU8	0.991832	0.402967	1.414442	0.536481	0.836431
DMU9	1	1	1	1	1
DMU10	0.855411	0.987798	0.805838	0.847758	0.874201
DMU11	0.904364	1.873826	0.798771	1.232885	1.202461
DMU12	0.025995	3.299138	14.22672	0.094406	4.411565
DMU13	0.900513	0.653923	1.060043	1.085883	0.925091
DMU14	1.045949	1.440275	0.981528	0.971161	1.109728

Average	**0.842245**	**3.337028**	**1.780453**	**0.766485**	**1.681553**

Max	1.180909	22.89828	14.22672	1.232885	6.183281

Min	0.025995	0.402967	0.093885	0.094406	0.731975

SD	0.350521	5.954769	3.595678	0.355252	1.618795

**Table 15:** Technical (frontier) change over the period from March 2010 to March 2014.

Frontier	March 10 → March 11	March 11 → March 12	March 12 → March 13	March 13 → March 14	Average
DMU1	1.207458	1.021252	1.216099	1.16575	1.15264
DMU2	1.004325	1.043617	1.058507	1.021122	1.031893
DMU3	1.590877	1.118066	2.701765	1.171436	1.645536
DMU4	3.799089	0.011946	1.037627	0.95957	1.452058
DMU5	1.014585	0.824552	1.537339	1.455841	1.208079
DMU6	0.942986	0.980633	1.144505	1.207582	1.068926
DMU7	1.071708	1.070017	1.059361	1	1.050272
DMU8	1.271446	0.699436	1.784794	1.45195	1.301906
DMU9	1	1	0.969405	0.834357	0.950941
DMU10	1.188513	1.137741	1.317842	1.222251	1.216587
DMU11	0.966224	1.10422	1.005262	0.777175	0.96322
DMU12	7.285402	0.882668	1.154549	0.65796	2.495145
DMU13	0.932239	1.085355	0.938112	0.931512	0.971805
DMU14	1.011873	0.947945	0.974758	1.011269	0.986461

Average	**1.734766**	**0.923389**	**1.278566**	**1.061984**	**1.249676**

Max	7.285402	1.137741	2.701765	1.455841	2.495145

Min	0.932239	0.011946	0.938112	0.65796	0.950941

SD	1.760654	0.289611	0.47398	0.233467	0.411515

**Table 16:** Productivity index (Malmquist-MPI) change over the period from March 2010 to March 2014.

Malmquist	March 10 → March 11	March 11 → March 12	March 12 → March 13	March 13 → March 14	Average
DMU1	1.024883	8.219062	0.114174	0.450252	2.452093
DMU2	0.962645	0.989855	1.05067	0.93656	0.984933
DMU3	1.860944	2.485255	1.211146	1.060732	1.654519
DMU4	0.32125	0.273534	0.799309	0.940345	0.58361
DMU5	0.836869	0.845502	0.965677	0.945678	0.898432
DMU6	1.11358	0.89969	0.807755	0.149455	0.74262
DMU7	1.071708	1.070017	1.059361	1	1.050272
DMU8	1.261062	0.281849	2.524487	0.778944	1.211586
DMU9	1	1	0.969405	0.834357	0.950941
DMU10	1.016668	1.123858	1.061967	1.036173	1.059666
DMU11	0.873818	2.069116	0.802974	0.958167	1.176019
DMU12	0.189382	2.912044	16.42545	0.062116	4.897249
DMU13	0.839494	0.709739	0.994439	1.011513	0.888796
DMU14	1.058368	1.365301	0.956752	0.982105	1.090631

Average	**0.959334**	**1.731773**	**2.124541**	**0.796171**	**1.402955**

Max	1.860944	8.219062	16.42545	1.060732	4.897249

Min	0.189382	0.273534	0.114174	0.062116	0.58361

SD	0.391846	2.017964	4.146581	0.330229	1.102699

**Table 17:** MPI change over the forecasted period from 2014 to 2018.

Malmquist	14 → 15	15 → 16	16 → 17	17 → 18	Average
DMU1	1.341873	0.849597	0.859625	0.862793	0.978472
DMU2	1.123411	1.075266	1.088532	1.097186	1.096098
DMU3	1.730123	1.175092	1.175136	1.175188	1.313884
DMU4	0.995231	1.029094	1.025264	1.018102	1.016923
DMU5	1.025688	1.008936	0.997077	0.996517	1.007055
DMU6	2.6849	1.405949	18.96484	4.671134	6.931705
DMU7	1.059873	1.049888	1.049889	1.049888	1.052384
DMU8	1.100092	1.001078	0.999625	0.997695	1.024622
DMU9	0.777466	0.900796	1	1	0.919565
DMU10	1.079077	1.064926	1.065922	1.066035	1.06899
DMU11	1.224279	1.021623	1.003503	1.004689	1.063524
DMU12	2.305589	1.261401	1.217948	1.163972	1.487227
DMU13	5.408089	0.832925	1.06676	1.786347	2.27353
DMU14	2.124731	0.65637	0.650852	0.647574	1.019882

Average	**1.712887**	**1.023782**	**2.297498**	**1.32408**	**1.589562**

Max	5.408089	1.405949	18.96484	4.671134	6.931705

Min	0.777466	0.65637	0.650852	0.647574	0.919565

SD	1.206086	0.185656	4.799086	0.993922	1.576408

**Table 18:** Summary of DMU's rankings over periods.

Ranking position	Past-present (2010–2014)	Future (2015–2018)
1	DMU4	DMU4
2	DMU2	DMU2
3	DMU11	DMU11
4	DMU13	DMU7
5	DMU7	DMU9
6	DMU9	DMU3
7	DMU14	DMU10
8	DMU6	DMU6
9	DMU3	DMU13
10	DMU8	DMU5
11	DMU12	DMU12
12	DMU10	DMU8
13	DMU1	DMU14
14	DMU5	DMU1

## References

[1] Reddy B. S., Ray B. K. (2010). Decomposition of energy consumption and energy intensity in Indian manufacturing industries. *Energy for Sustainable Development*.

[2] Grover R. B., Chandra S. (2006). Scenario for growth of electricity in India. *Energy Policy*.

[3] Muneer T., Asif M., Munawwar S. (2005). Sustainable production of solar electricity with particular reference to the Indian economy. *Renewable and Sustainable Energy Reviews*.

[4] Ghosh S. (2002). Electricity consumption and economic growth in India. *Energy Policy*.

[5] Wolde-Rufael Y. (2006). Electricity consumption and economic growth: a time series experience for 17 African countries. *Energy Policy*.

[6] Ferguson R., Wilkinson W., Hill R. (2000). Electricity use and economic development. *Energy Policy*.

[7] Sun C. C. (2011). Assessing Taiwan financial holding companies performance using window analysis and Malmquist productivity index. *African Journal of Business Management*.

[8] Deng J. L. (1989). Introduction to grey system theory. *The Journal of Grey System*.

[9] Filippini M., Pachauri S. (2004). Elasticities of electricity demand in urban Indian households. *Energy Policy*.

[10] Sharma D. P., Nair P. S. C., Balasubramanian R. (2005). Performance of Indian power sector during a decade under restructuring: a critique. *Energy Policy*.

[11] Thakur T., Deshmukh S. G., Kaushik S. C. (2006). Efficiency evaluation of the state owned electric utilities in India. *Energy Policy*.

[12] Zhou P., Ang B. W., Poh K. L. (2008). A survey of data envelopment analysis in energy and environmental studies. *European Journal of Operational Research*.

[13] Lo S.-F., Lu W.-M. (2009). An integrated performance evaluation of financial holding companies in Taiwan. *European Journal of Operational Research*.

[14] Kao L.-J., Chiu S.-Y., Ko H.-T. (2014). A study of the talent training project management for semiconductor industry in Taiwan: the application of a hybrid data envelopment analysis approach. *The Scientific World Journal*.

[15] Deng J.-L. (1981/82). Control problem of grey systems. *Systems & Control Letters*.

[16] Huang Y.-P., Yu T.-M. (1997). The hybrid grey-based models for temperature prediction. *IEEE Transactions on Systems, Man, and Cybernetics Part B: Cybernetics*.

[17] An S., Yan J., Yu X. (1996). Grey-system studies on agricultural ecoengineering in the Taihu lake area, Jiangsu, China. *Ecological Engineering*.

[18] Wang C. N., Nguyen N. T. (2013). Forecasting the manpower requirement in Vietnamese tertiary institutions. *Asian Journal of Empirical Research*.

[19] Farrell M. J. (1957). The measurement of productive efficiency. *Journal of the Royal Statistical Society A: General*.

[20] Ittner C. D., Larcker D. F. (1998). Are nonfinancial measures leading indicators of financial performance? An analysis of customer satisfaction. *Journal of Accounting Research*.

[21] Baier C., Hartmann E., Moser R. (2008). Strategic alignment and purchasing efficacy: an exploratory analysis of their impact on financial performance. *Journal of Supply Chain Management*.

[22] Simpson W. G., Kohers T. (2002). The link between corporate social and financial performance: evidence from the banking industry. *Journal of Business Ethics*.

[23] Fare R., Grosskopf S., Norris M. (1994). Productivity growth, technical progress, and efficiency change in industrialized countries. *The American Economic Review*.

[24] Tone K. (2001). A slacks-based measure of efficiency in data envelopment analysis. *European Journal of Operational Research*.

[25] Tone K. (2002). A slacks-based measure of super-efficiency in data envelopment analysis. *European Journal of Operational Research*.

[26] Stevenson J. W. (2009). *Operations Management*.

[27] Lo F.-Y., Chien C.-F., Lin J. T. (2001). A DEA study to evaluate the relative efficiency and investigate the district reorganization of the Taiwan Power Company. *IEEE Transactions on Power Systems*.

[28] Boles J. S., Donthu N., Lohtia R. (1995). Salesperson evaluation using relative performance efficiency: the application of data envelopment analysis. *Journal of Personal Selling & Sales Management*.

[29] Helleiner E. (2011). Understanding the 2007-2008 global financial crisis: lessons for scholars of international political economy. *Annual Review of Political Science*.

